# BLM helicase inhibition synergizes with PARP inhibition to improve the radiosensitivity of olaparib resistant non-small cell lung cancer cells by inhibiting homologous recombination repair

**DOI:** 10.20892/j.issn.2095-3941.2021.0178

**Published:** 2021-12-01

**Authors:** Yangyang Kong, Chang Xu, Xiaohui Sun, Hao Sun, Xiaotong Zhao, Ningning He, Kaihua Ji, Qin Wang, Liqing Du, Jinhan Wang, Manman Zhang, Yang Liu, Yan Wang, Qiang Liu

**Affiliations:** 1 Tianjin Key Laboratory of Radiation Medicine and Molecular Nuclear Medicine, Institute of Radiation Medicine, Chinese Academy of Medical Sciences & Peking Union Medical College, Tianjin 300192, China

**Keywords:** NSCLC, PARP, BLM, radiosensitization, homologous recombination repair

## Abstract

**Objective:**

We aimed to investigate the radiosensitizing efficacy of the poly-ADP-ribose polymerase (PARP) inhibitor, olaparib, and the Bloom syndrome protein (BLM) helicase inhibitor, ML216, in non-small cell lung cancer (NSCLC) cells.

**Methods:**

Radiosensitization of NSCLC cells was assessed by colony formation and tumor growth assays. Mechanistically, the effects of ML216, olaparib, and radiation on cell and tumor proliferation, DNA damage, cell cycle, apoptosis, homologous recombination (HR) repair, and non-homologous end joining (NHEJ) repair activity were determined.

**Results:**

Both olaparib and ML216 enhanced the radiosensitivities of olaparib-sensitive H460 and H1299 cells, which was seen as decreased surviving fractions and Rad51 foci, increased total DNA damage, and γH2AX and 53BP1 foci **(***P* < 0.05). The expressions of HR repair proteins were remarkably decreased in olaparib-treated H460 and H1299 cells after irradiation (*P* < 0.05), while olaparib combined with ML216 exerted a synergistic radiosensitization effect on olaparib-resistant A549 cells. In addition to increases of double strand break (DSB) damage and decreases of Rad51 foci, olaparib combined with ML216 also increased pDNA-PKcs (S2056) foci, abrogated G2 cell cycle arrest, and induced apoptosis in A549 lung cancer after irradiation *in vitro* and *in vivo* (*P* < 0.05). Moreover, Western blot showed that olaparib combined with ML216 and irradiation inhibited HR repair, promoted NHEJ repair, and inactivated cell cycle checkpoint signals both *in vitro* and *in vivo* (*P* < 0.05).

**Conclusions:**

Taken together, these results showed the efficacy of PARP and BLM helicase inhibitors for radiosensitizing NSCLC cells, and supported the model that BLM inhibition sensitizes cells to PARP inhibitor-mediated radiosensitization, as well as providing the basis for the potential clinical development of this combination for tumors intrinsically resistant to PARP inhibitors and radiotherapy.

## Introduction

Lung carcinomas are the leading cause of cancer-related deaths worldwide, with a 5-year overall survival of less than 15%^1,2^. Non-small cell lung cancer (NSCLC) is the most common histological type of lung cancer, accounting for approximately 85% of lung cancer cases, with 65% of the patients presenting with unresectable stage III and IV disease^3^. Currently, the main treatments for this disease are surgery, radiotherapy, chemotherapy, and targeted therapy^4,5^. NSCLC can occur as unusual histological variants, which represent a heterogeneous group of lung cancers, and thus are relatively insensitive to radiotherapy, when compared with small cell lung cancer and other cancers^6,7^. The therapeutic results are many times ineffective due to the limited maximal dose of irradiation and the emergence of radiochemoresistance that leads to local recurrence and distant metastasis. Development of efficient radiosensitizers is therefore a strategy for enhancing the response of NSCLC to radiotherapy.

The poly-ADP-ribose polymerase (PARP) family of enzymes are known as universal sensors of DNA damage, and are important players in DNA repair. They catalyze the polymerization of ADP-ribose to themselves and other DNA repair proteins at DNA damage sites^8–10^. PARP inhibitors are a class of anti-neoplastic agents that are being widely tested in homologous recombination (HR)-deficient solid tumors^11–13^. In addition to breast cancer susceptible gene (BRCA) deficiency, heightened sensitivity to PARP inhibition has also been observed in cells with other genetic lesions that affect HR, including phosphatase and tensin homolog (PTEN) deficiency, ataxia telangiectasia mutated (ATM) deficiency, and Aurora A overexpression^14–16^. Subsequent drug discovery efforts have promoted the development of clinical PARP inhibitors, including veliparib (Abbvie), rucaparib (Pfizer/Clovis), olaparib (KuDOS/AstraZeneca), and niraparib (Merck/Tesaro)^17^. The rationale for the identification of effective PARP inhibitor combinations has concentrated largely on enhancing the anti-tumor effect of PARP inhibitors by creating DNA damage or modulating DNA repair. The mechanisms by which PARP inhibitors enhance cellular radiosensitivity involve inhibiting PARP1-mediated repair of radiation-induced DNA damage and increasing tumor blood flow^18^. Other studies have shown that the PARP trapping activity of PARP inhibitors interfered with cellular replication^19^, and that PARP inhibitors enhanced tumor radiosensitivity by inducing replication-mediated double strand breaks (DSBs)^20^.

Bloom’s syndrome protein, designated BLM, is 1 of 5 conserved RecQ DNA helicase family members in human cells, along with RECQ1, RECQ4, RECQ5, and WRN^21^. DNA helicases are involved in all interactions with DNA that require the opening of the duplex, including DNA replication, RNA transcription, HR, and many other forms of DNA repair. Evidence has supported roles for BLM in multiple steps of HR-dependent DSB repair including 5′-end resection, Rad51 filament formation, DNA displacement loop formation, and double Holliday junction recombination intermediate structure dissolution^22–24^. The absence of functional BLM protein causes chromosome instability, excessive HR, and greatly increased numbers of sister chromatid exchanges that are the pathogenomic diagnoses of Bloom’s syndrome^25^. Recent studies have shown that degradation of BLM protein by miR-522-3p promoted tumorigenesis in human colorectal cancer^26^. There are few reports on BLM helicase inhibitors^27–30^, among which ML216 is the first and only commercial small molecule inhibitor, which acutely disables BLM function in human cells. ML216 modulates chromosome stability through competitive inhibition of the DNA binding activity of BLM, potently inhibiting the DNA unwinding activity of BLM and inducing increases of sister chromatid exchange. The analogs of ML216 or newly developed BLM inhibitors can be used clinically to target 5%–10% of the alternative lengthening of telomere tumors, such as osteosarcoma, which could provide a new therapeutic strategy for targeting these treatment-resistant tumors^27^. ML216 could therefore be useful to more specifically target BLM inactivation in further studies, and as a potential chemotherapeutic agent. However, studies of ML216 in the field of radiation and NSCLC have not yet been reported. Considering that BLM promotes HR repair, we comprehensively studied the radiosensitization of ML216 in NSCLC.

In this study, we investigated the cell proliferation inhibition and radiosensitization effects of olaparib and ML216 in 3 NSCLC cell lines (H460, H1299, and A549). Mechanistically, the inhibition of HR repair was critical for the radiosensitization of olaparib and ML216 in olaparib-sensitive H460 and H1299 cells. In addition, olaparib combined with ML216 exerted a synergistic radiosensitization effect on olaparib-resistant A549 cancers *in vitro* and *in vivo*, which was mediated by inhibiting HR repair, abrogating G2 cell cycle arrest, and ultimately inducing apoptosis.

## Materials and methods

### Reagents

Roswell Park Memorial Institute (RPMI) 1,640 culture medium was purchased from HyClone (Logan, UT, USA). Fetal bovine serum (FBS) was purchased from Gibco (Grand Island, NY, USA). Olaparib (AZD2281, Ku-0059436) was purchased from Selleck (Houston, TX, USA). ML216 (CID-49852229) was purchased from MedChemExpress (Monmouth Junction, NJ, USA). Antibodies against pBRCA1 (Ser1524), p53BP1 (Ser1778), pATM (Ser1981), pATR (Ser428), Rad50, Mre11, pDNA-PKcs (Ser2056), Ku80, pChk2 (Thr68), pChk1 (Ser317), pRb (Ser807/811), pRb (Ser795), p21, and γH2AX were purchased from Cell Signaling Technology (Danvers, MA, USA). Antibodies against RPA70, Rad51, 53BP1, CyclinA, and Ki-67 were purchased from Abcam (Cambridge, UK). Antibodies against β-tubulin, β-actin, and glyceraldehyde 3-phosphate dehydrogenase were purchased from Proteintech (Rosemont, IL, USA).

### Cell culture

The NSCLC cell lines (H460, H1299, and A549) were purchased from the American Type Culture Collection (Manassas, VA, USA). NSCLC cells were grown in RPMI 1,640 medium supplemented with 10% FBS at 37 °C with 5% CO_2_. Olaparib and ML216 were dissolved in dimethyl sulfoxide (DMSO) at stock concentrations of 5 mM and 10 mM. All samples in any given cell experiment contained identical concentrations of DMSO (0.1% v/v).

### Cell irradiation

An irradiator equipped with a Cs-137 (Gammacell-40) source was purchased from Atomic Energy of Canada (Mississauga, ON, Canada). Sealed, sterile cell culture plates or dishes were placed in the center of the irradiation chamber and exposed to irradiation at a dose of 1.02 Gy/min.

### MTT cell viability assay

Cells were inoculated into 96-well plates with the indicated concentrations of olaparib or ML216 for 3 or 5 days. Twenty µL of 3-(4,5-dimethylthiazol-2-yl)-2,5-diphenyl tetrazolium bromide (MTT) at 5 mg/mL was added to each well, and incubated for 3 h. The absorbance was measured at 492 nm using a microplate reader. Each drug concentration was conducted using 6 replicate wells, and the experiment was repeated at least 3 times. Cell survival was expressed as the absorbance of each condition relative to that of untreated controls.

### Colony formation assay

NSCLC cells (H460, H1299, and A549) were seeded at 1,000 cells per well in a 6-well plate in triplicate and then incubated for 24 h to allow adhesion. NSCLC cells were γ-irradiated and maintained for 10–14 days before staining with 0.25% Crystal Violet in ethanol. Colonies containing more than 50 cells were counted. The surviving fraction of irradiated cells was normalized to the plating efficiency of non-irradiated controls. The sensitizer enhancement ratios at 37% survival were calculated using a classical multi-target single-hit model using the following equation:

(1) $$S(D)=1-{\left(1-e^{-D/D_{37}}\right)}^{n}$$

(2) $$SER_{37}=\frac{D_{37}(no\;drug)}{D_{37}(drug)}$$

Where *S*(*D*) is the survival fraction, *D* is the radiation dose, *D*_37_ is the lethal dose at 37% survival, and n is the extrapolation number. In the above model formula, *D*_37_ and *n* were calculated using Prism 5.0 software (GraphPad, San Diego, CA, USA).

### The alkaline single cell gel electrophoresis assay (alkaline comet assay)

Olaparib (5 µM) or ML216 (10 µM) was administered 3 days before 4 Gy of irradiation. After irradiation, the cells were lysed immediately in cold fresh lysis solution for 2.5 h at 4 °C. After lysis, the cells were electrophoresed and neutralized. The dyed comet slides were viewed with a fluorescence microscope, and data were collected with a digital imaging system. At least 100 cells were analyzed using CASP software (https://bio.tools/casp).

### Cellular immunofluorescence assay

Cells with appropriate densities were treated with DMSO/inhibitors before irradiation for 2 days. At 24 h after irradiation, the cells grown on cover slides were washed twice with cold phosphate-buffered saline (PBS) and fixed with 4% paraformaldehyde/PBS for 30 min at room temperature. Fixed cells were permeabilized with 0.3% Triton X-100/PBS for 30 min at room temperature. The cells were then incubated with primary antibodies overnight at 4 °C. After being washed 3 times with PBS, the cells were incubated for 1 h with Cy3-conjugated anti-rabbit IgG secondary antibody. The nuclei were counterstained with 4′-6-diamidino-2-phenylindole. Immunofluorescence was observed and photographed using a fluorescence microscope. Cells with more than 10 discrete foci per cell were regarded as positive cells, and at least 300 cells were manually scored for quantitation.

### Cell cycle analysis

Cells were harvested by trypsinization, washed in cold PBS, and fixed with cold 70% ethanol overnight at 4 °C. After washing, the DNA was stained with propidium iodide (PI) solution containing RNase A. Cell cycle distribution was monitored by flow cytometry (FACScan; BD Biosciences, San Jose, CA, USA) and analyzed using Flow Jo software (https://www.flowjo.com/).

### Apoptosis assay

Apoptosis was measured by fluorescence-activated cell sorting using the Annexin V-FITC Apoptosis Detection Kit (BD Biosciences). In brief, A549 cells were collected and washed with PBS, then stained with annexin V-FITC/PI. The samples were analyzed using flow cytometry.

### Protein extraction and Western blot

Whole cell lysates were isolated using protein extract buffer, and tumor proteins were isolated using RIPA lysates (Solarbio, Beijing, China) with phenylmethylsulfonyl fluoride (PMSF) in the cocktail. Equal amounts of total protein were electrophoresed using SDS/PAGE. Protein signals were visualized using chemiluminescence reagents. Protein concentrations were estimated by the BCA method. The protein expression was analyzed by ImageJ Lab software (Bio-Rad, Hercules, CA, USA).

### *In vivo* xenograft studies

BABL/c Nu/Nu male nude mice were obtained from Beijing Huafukang Biotechnology (Beijing, China) and housed under standard laboratory conditions. A549 cells (10 × 10^6^) were subcutaneously injected into the buttocks of 7-week-old mice. Mice (*N* = 5–10 mice/group) with A549 xenografts were treated with DMSO (4%), olaparib (25 mg/kg), or ML216 (25 mg/kg) for 3 continuous days. X-ray irradiation (1.22 Gy/min dose, 8 Gy) was given on the second day of administration. All animal procedures complied with the guidelines of our local animal care and use committee (Approval No. IRM-DWLL-2018106).

### Immunohistochemistry staining

Tissue sections were deparaffinized and hydrated. Antigen retrieval was performed in pH 6.0 citrate buffer and washed using TBST. Peroxide blocking was performed at ambient temperatures for 20 min using 3% H_2_O_2_ in distilled water. The slides were incubated with primary Ki-67 antibody overnight at 4 °C and washed with TBST buffer, followed by incubation with biotin-labeled secondary antibody for 20 min. Staining was developed with fresh 3,3′-diaminobenzidine for 30s and then counterstained with hematoxylin, dehydrated, and mounted. Images were visualized using an upright microscope. The percentage of positive cells was used to evaluate the expression of Ki-67.

### TdT-UTP nick-end labeling (TUNEL)

TUNEL assays were performed with a one step TUNEL apoptosis assay kit (Beyotime, Beijing, China). This method took advantage of DNA fragmentation, which is characteristic of apoptosis. Immunohistochemical procedures for detecting apoptotic A549 tumor cells were performed according to the manufacturer’s instructions. At least 5 fields of each section were randomly chosen using a fluorescence microscope. The percentage of apoptosis was determined (i.e., number of positively stained apoptotic cells/total number of cells counted × 100). Assays were performed in a blinded manner.

### Statistical analysis

Statistical analysis was performed using SPSS statistical software for Windows (SPSS, Chicago, IL, USA). Data are presented as the mean *±* SEM. Each experiment was performed at least 3 times. A 2-tailed Student’s *t*-test was used to measure the difference between the 2 groups. The differences between more than 2 groups were analyzed for significance using one-way analysis of variance. *P*-values < 0.05 were considered statistically significant. Data are representative of 3 independent experiments.

## Results

### Olaparib combined with ML216 enhances the radiosensitivity of olaparib-resistant A549 cells

**Figure 1:**
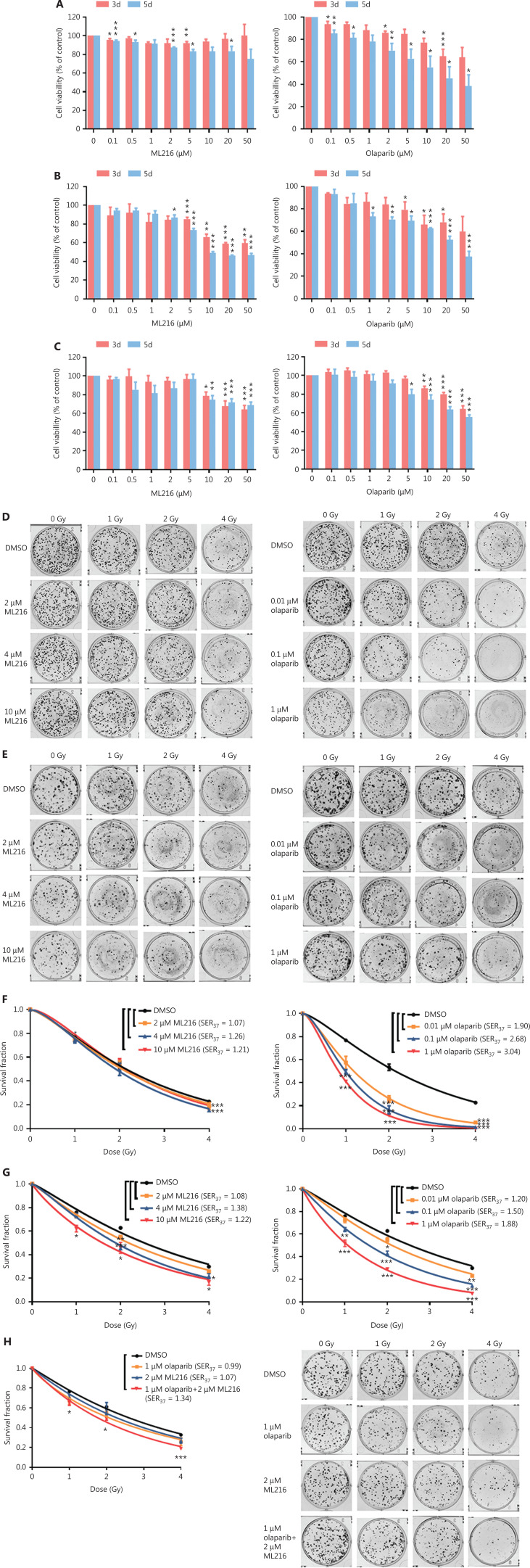
Olaparib and ML216 selectively sensitize NSCLC cell lines to irradiation. (A–C) Cell viability was determined using the MTT assay and expressed as a percentage of the viability of untreated cells. NSCLC cells (H460, H1299, and A549) were exposed to increasing concentrations of olaparib or ML216 for 3 or 5 days. (D–H) Cells were treated with dimethyl sulfoxide, olaparib, or ML216 prior to exposure to the indicated doses of irradiation. The survival fractions were then measured after 10–14 days. Shown are the means *±* SEM from 3 experiments (*P < 0.05; **P < 0.01; ***P < 0.005). NSCLC, non-small cell lung cancer.

Before evaluating the abilities of olaparib and ML216 to radiosensitize NSCLC cells, we first detected the sensitivities of 3 NSCLC cell lines (H1299, H460, and A549) to olaparib and ML216 in the absence of irradiation. Three NSCLC cell lines were exposed to increasing concentrations of olaparib or ML216, and the cell viability was analyzed using MTT assays. When comparing the cell viabilities, we found that both olaparib and ML216 produced a concentration-dependent cell proliferation inhibition of H1299, H460, and A549 cells (*P* < 0.05). The sensitivities to olaparib or ML216 differed (H460 > H1299 > A549 for olaparib and H1299 > A549 > H460 for ML216) (**Figure 1A–C**). Compared with H460 and H1299 cells, the IC_50_ values of the inhibitors showed that A549 cells were most resistant to olaparib (**Supplementary Table S1**). Next, we detected the radiosensitization effect of olaparib and ML216 on the 3 NSCLC cell lines using a clonogenic survival assay. According to the cell proliferation inhibition effect using the MTT assay, we chose non-toxic concentrations of inhibitors, including 0.01–1 µM olaparib and 2–10 µM ML216. Corresponding to the sensitivity of cells to olaparib, even a low concentration (0.01 µM) of olaparib significantly decreased surviving fractions of H460 and H1299 cells (*P* < 0.05), and enhanced the radiosensitivity of H460 and H1299 cells (**Figure 1D–G**). Similarly, ML216 significantly decreased surviving fractions of H1299 cells in a dose-dependent manner (*P* < 0.05), and enhanced the radiosensitivity of H1299 cells (**Figure 1E, 1G**). Although ML216 had a slight inhibitory effect on H460 cells, ML216 significantly enhanced the radiosensitivity of H460 cells (*P* < 0.05) (**Figure 1D, 1F**). More importantly, olaparib combined with ML216 significantly reduced surviving fractions and enhanced the radiosensitivity of A549 cells relative to either agent alone (**Figure 1H**), suggesting that olaparib combined ML216 exerted a synergistic radiosensitization effect on olaparib-resistant A549 cells.

### Olaparib and ML216 enhance total DNA damage and delay DSB repair in NSCLC cells after irradiation

**Figure 2:**
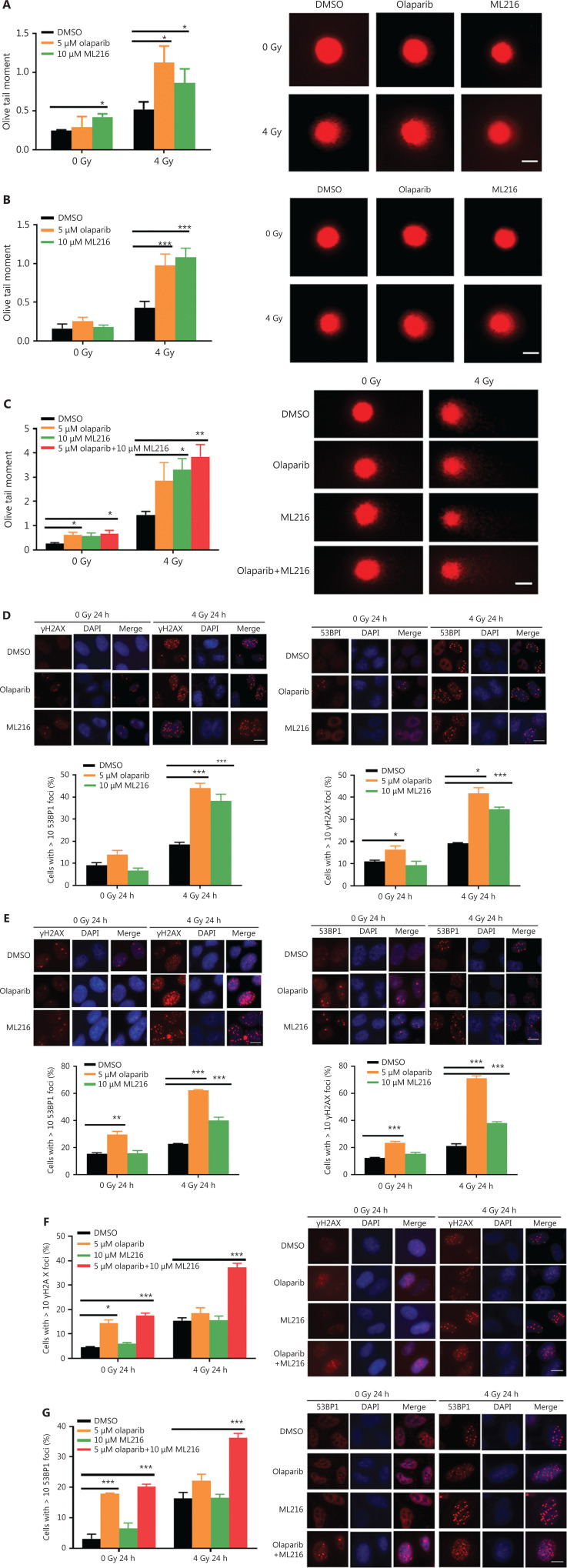
Olaparib and ML216 enhance total DNA damage, and delay double strand break repair in non-small cell lung cancer (NSCLC) cells. (A-C) NSCLC cells (H460, H1299, and A549) treated with dimethyl sulfoxide (DMSO), 5 μM olaparib, or 10 μM ML216 were immediately lysed after 4 Gy of irradiation. The olive tail moment was used as an index and was statistically analyzed. At least 100 cells were analyzed using CASP software. (D-G) NSCLC cells (H460, H1299, and A549) treated with DMSO, 5 μM olaparib, or 10 μM ML216 were fixed in polyformaldehyde for 24 h, after 4 Gy of irradiation. The cells with more than 10 foci per cell were regarded as positive cells, and at least 300 cells were counted. The scale bars represent 25 μm. The ratio of positive cells and representative pictures are shown. Shown are the means ± SEM from 3 experiments (*P < 0.05; **P < 0.01; ***P < 0.005).

Exogenous and endogenous DNA targeting exposures, such as chemical toxins and irradiation, induce DNA damage responses (DDR) that include DNA damage repair, cell cycle arrest, and apoptosis. To determine the effects of olaparib and ML216 on irradiation-induced DNA damage, total DNA lesions were assessed using an alkaline comet assay with an olive tail moment as the detection index. Compared with irradiation alone, the olive tail moment was significantly increased in olaparib- or ML216-treated H460 and H1299 cells after irradiation (*P* < 0.05) (**Figure 2A–B**), while in A549 cells, olaparib combined with ML216 significantly increased total DNA lesions with or without irradiation (*P* < 0.05) (**Figure 2C**). DNA DSBs are considered to be the most toxic DNA damage, causing cell death, and induced by irradiation during radiotherapy^31^. We evaluated the effects of olaparib and ML216 on DSB repair by measuring γH2AX and 53BP1 foci formation used as surrogate DSB markers^32^. Both olaparib and ML216 significantly increased γH2AX and 53BP1 foci formation in H460 and H1299 cells after irradiation (*P* < 0.05) (**Figure 2D–E**). In addition, olaparib combined with ML216 significantly increased γH2AX and 53BP1 foci formation of A549 cells in the absence or presence of irradiation (*P* < 0.05) (**Figure 2F–G**). These results indicated that olaparib and ML216 caused persistently impaired DSB repair, which could be one of mechanisms of radiosensitization of NSCLC cells.

### ML216 enhances the inhibitory effect of olaparib on HR repair in A549 cells after irradiation

**Figure 3:**
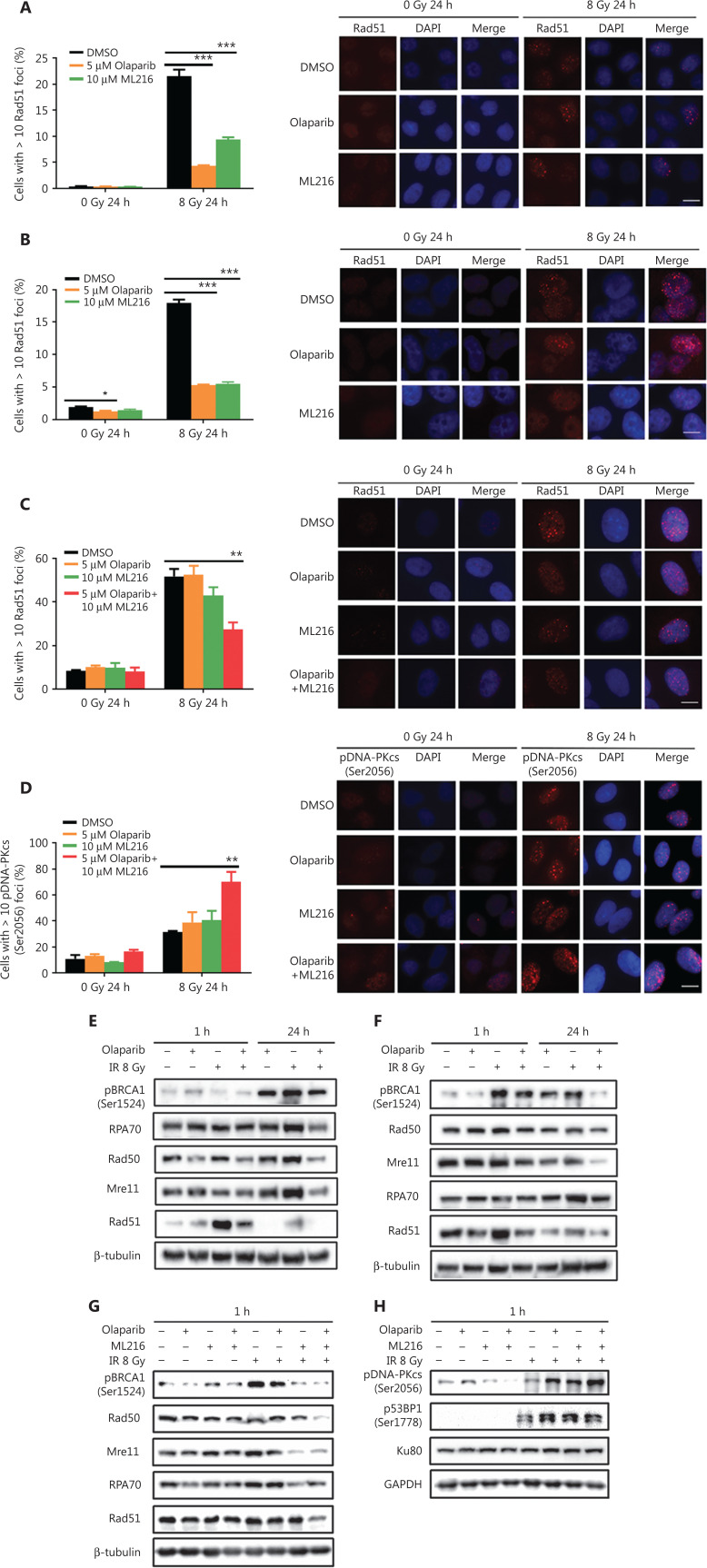
Olaparib and ML216 dysregulate double strand break repair of non-small cell lung cancer (NSCLC) cells in response to irradiation. (A–D) NSCLC cells (H460, H1299, and A549) treated with dimethyl sulfoxide (DMSO), 5 μM olaparib, or 10 μM ML216 were fixed in paraformaldehyde for 24 h, after 8 Gy of irradiation. The cells with more than 10 foci per cell were regarded as positive cells, and at least 300 cells were counted. The ratio of positive cells and representative pictures are shown. (E–H) NSCLC cells (H460, H1299, and A549) treated with DMSO, 5 μM olaparib, or 10 μM ML216 were harvested at 1 h or 24 h, after 8 Gy of irradiation. The cells were lysed using protein extraction buffer and then denatured at 100 °C for 10 min. The protein samples were resolved using SDS-PAGE and analyzed by ImageJ Lab software. Representative pictures of Western blot are shown. Shown are the means *±* SEM from 3 experiments (*P < 0.05; **P < 0.01; ***P < 0.005).

DSBs are mainly repaired either by HR or by non-homologous end joining (NHEJ) pathways^33,34^. We determined which repair pathways were specifically affected by olaparib and ML216. Rad51 foci and pDNA-PKcs (Ser2056) foci were used as surrogate markers of HR and NHEJ repairs, respectively^35,36^. A cellular immunofluorescence assay showed that both olaparib and ML216 significantly decreased Rad51 foci formation in H460 and H1299 cells after irradiation (*P* < 0.05) (**Figure 3A–B**). Consistently, Western blot showed that expressions of several critical HR repair proteins, including pBRCA1 (Ser1524), Rad50, Mre11, RPA70, and Rad51^37,38^, were decreased in olaparib-treated H460 and H1299 cells after irradiation (*P* < 0.05) (**Figure 3E–F and Supplementary Figure S1**). These results indicated that both olaparib and ML216 enhanced the radiosensitivities of H460 and H1299 cells by inhibiting HR repair. In addition, we detected pDNA-PKcs (Ser2056) foci formation in H1299 cells. The results showed that both olaparib and ML216 increased pDNA-PKcs (Ser2056) foci formation in H1299 cells after irradiation (**Supplementary Figure S2**), suggesting that both olaparib and ML216 induced the radiosensitivity of H1299 cells by promoting NHEJ repair. Furthermore, we characterized the possible mechanism of the radiosensitization effect of olaparib combined with ML216 on A549 cells. In A549 cells, olaparib combined with ML216 significantly decreased Rad51 foci after irradiation (**Figure 3C**). In contrast, olaparib combined with ML216 increased pDNA-PKcs (Ser2056) foci of A549 cells after irradiation (*P* < 0.05) (**Figure 3D**). Consistently, Western blot showed that olaparib combined with ML216 decreased protein expressions of pBRCA1 (Ser1524), Rad50, Mre11, RPA70, and Rad51 in A549 cells after irradiation (*P* < 0.05) (**Figure 3G and Supplementary Figure S3**), while expressions of crucial NHEJ repair proteins, including pDNA-PKcs (Ser2056), p53BP1 (Ser1778), and Ku80^39^, were increased in olaparib and ML216 combination-treated A549 cells following irradiation (*P* < 0.05) (**Figure 3H and Supplementary Figure S3**). These results suggested that olaparib combined with ML216 produced a synergistic radiosensitization effect by inhibiting error-free HR repair and promoting error-prone NHEJ repair in olaparib-resistant A549 cells.

### Olaparib combined with ML216 abrogates G2 cell cycle arrest and induces apoptosis in A549 cells after irradiation

**Figure 4:**
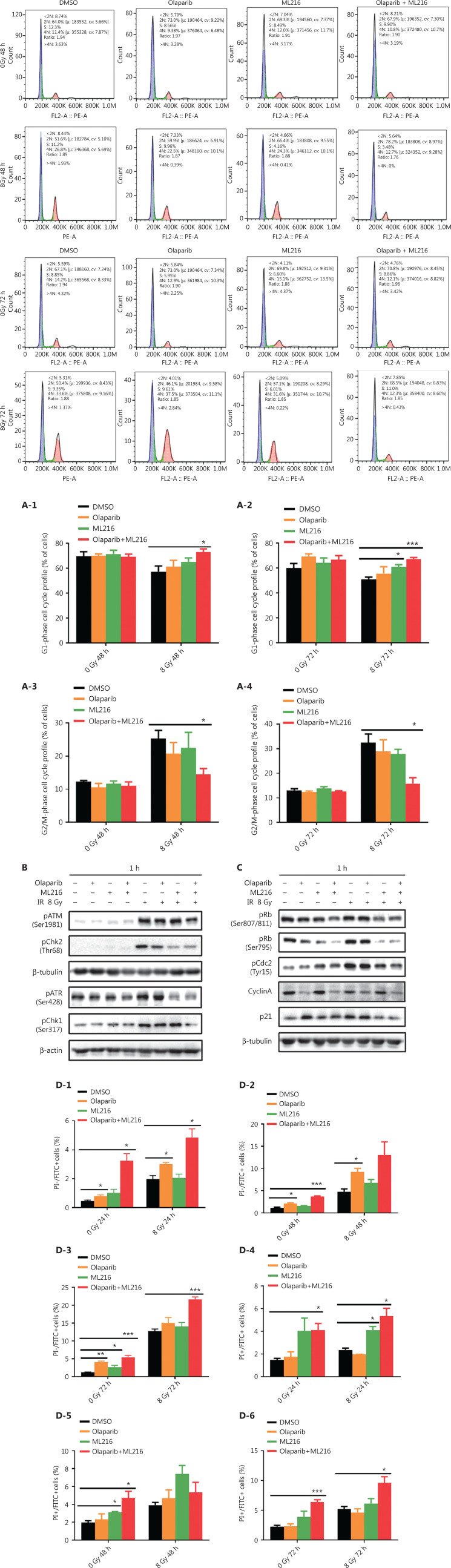
Olaparib combined with ML216 abrogated G2 cell cycle arrest and induced apoptosis of A549 cells after irradiation. (A) A549 cells treated with dimethyl sulfoxide (DMSO), 5 μM olaparib, or 10 μM ML216 were collected 48 h and 72 h, after 8 Gy of irradiation. The cells were fixed in 70% ethanol and detected by flow cytometry. Distributions of the cell cycle were statistically analyzed and representative pictures are shown. (B–C) A549 cells treated with DMSO, 5 μM olaparib, or 10 μM ML216 were harvested at 1 h after 8 Gy of irradiation. The cells were lysed in protein extraction buffer and then denatured at 100 °C for 10 min. The protein samples were resolved by SDS-PAGE and analyzed by ImageJ Lab software. Representative pictures of Western blot are shown. (D) A549 cells added to DMSO, 5 μM olaparib, or 10 μM ML216 were detected using an apoptosis detection kit after flow cytometry at 24 h, 48 h, or 72 h, after 8 Gy of irradiation. Shown are the means ± SEM from 3 experiments (*P < 0.05; **P < 0.01; ***P < 0.005).

We next focused on the radiosensitization mechanism of olaparib combined with ML216 of olaparib-resistant A549 cells. In response to DNA damage, cells activate cell cycle checkpoints that prevent further progression through the cell cycle. Correspondingly, DNA repair pathways are implemented in a cell cycle dependent manner, in which HR repair functions only in S/G2 phases and NHEJ functions dominantly in G1 phases^36,40^. Flow cytometric analysis of PI-stained cells revealed that olaparib combined with ML216 increased the G1 cell content and decreased the G2/M cell content in A549 cells after irradiation (*P* < 0.05) (**Figure 4A**). Traditionally, 2 major kinase pathways, ATM/Chk2 and ATR/Chk1, are activated in response to DNA DSBs and DNA single-strand breaks or bulky lesions, which function upstream of checkpoint signaling^41^. Western blot showed that olaparib combined with ML216 significantly inhibited phosphorylation of ATM (Ser1981)-Chk2 (Thr68) and ATR (Ser428)-Chk1 (Ser137) in A549 cells after irradiation (*P* < 0.05), suggesting that olaparib combined with ML216 impeded DNA damage repair and inhibited activation of checkpoint signaling (**Figure 4B and Supplementary Figure S3**). We also found that olaparib combined with ML216 significantly inhibited S/G2 phase-specific accumulation of CyclinA, consistent with the inhibition of G2 cell cycle arrest (*P* < 0.05) (**Figure 4C and Supplementary Figure S3**). Phosphorylation of Cdc2 (Tyr15) is a critical event in the G2/M checkpoint, and Cdc2 is also a major pRb kinase^42,43^. Consistent with the inhibition of G2 cell cycle arrest, olaparib combined with ML216 significantly decreased the phosphorylation of Cdc2 (Tyr15), Rb (Ser807/811), and Rb (Ser795) in A549 cells after irradiation (*P* < 0.05) (**Figure 4C and Supplementary Figure S3**). The cell cyclin-dependent kinase inhibitor, p21, promotes cell cycle arrest in response to γ-irradiation and is essential for G2 arrest^44^. Western blot showed that olaparib combined with ML216 decreased the protein level of p21 in A549 cells after irradiation (*P* < 0.05) (**Figure 4C and Supplementary Figure S3**). Furthermore, annexin V binding assays were used to evaluate apoptotic cell death, including early apoptosis (PI^−^/FITC^+^), late apoptosis, and necrosis (PI^+^/FITC^+^). Olaparib combined with ML216 significantly increased total apoptosis in the absence or presence of irradiation (*P* < 0.05) (**Figure 4D and Supplementary Figure S4**). These results demonstrated that the inhibition of G2 cell cycle arrest and induction of apoptosis were critical mechanisms of the radiosensitization effect of olaparib combined with ML216 on olaparib-resistant A549 cells.

### The radiosensitization effect of olaparib combined with ML216 on A549 xenografts *in vivo*

**Figure 5:**
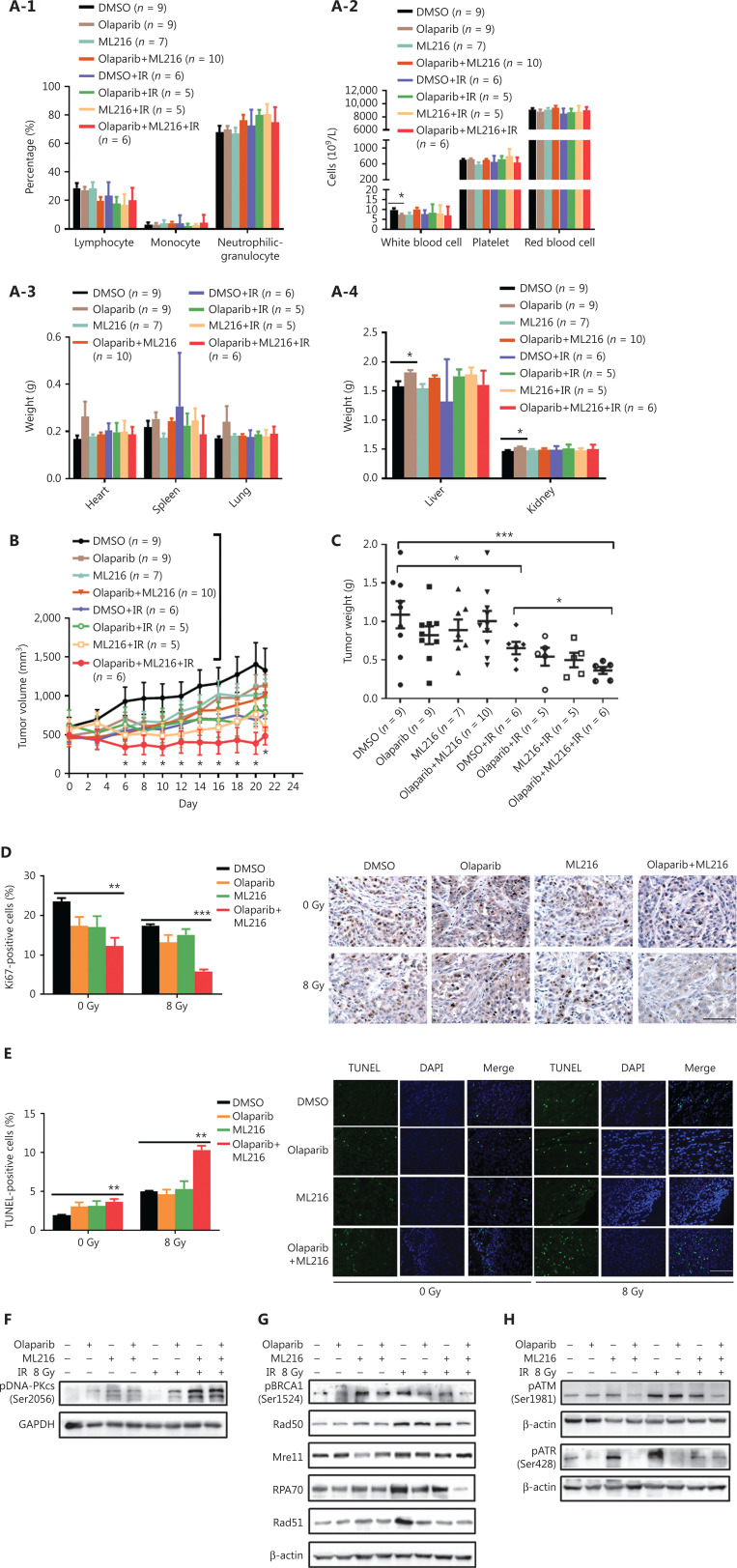
Olaparib synergizes with ML216 effects against A549 non-small cell lung cancer *in vivo*. (A) After the mice were sacrificed, the main organs were weighed and blood was analyzed. The blood cells and main organs were statistically analyzed and the results are shown as a histogram. (B) Tumor volume was measured once every 2 days. After 21 days, the mice were sacrificed, and the tumors were removed and analyzed. The tumor volume used as an index of growth is shown. (C) The tumor weight used as an index of radiosensitivity was analyzed and a representative picture is shown. (D) Ki-67 tumor tissues were analyzed by immunohistochemical staining and quantified by ImageJ software. The ratio of Ki-67 positive cells is shown using a histogram. (E) A TUNEL apoptosis assay kit was used to detect apoptosis in paraffin sections of A549 xenograft tumors. The ratio of apoptotic cells is shown using a histogram. (F–H) The A549 tumor samples were stored at -80 °C and lysed in RIPA buffer containing phenylmethylsulfonyl fluoride and protease inhibitors. The protein samples were resolved by SDS-PAGE and analyzed by ImageJ Lab software. Representative pictures of Western blot are shown. The data represent the means ± SEM from 3 experiments (*P < 0.05; **P < 0.01; ***P < 0.005).

We finally sought to confirm the radiosensitization effect of olaparib and ML216 *in vivo*. Nude mice bearing A549-derived xenografts were treated with DMSO, olaparib (25 mg/kg/d), or ML216 (25 mg/kg/d) for 3 days, and irradiation was given on the second day of administration. We first detected normal tissue toxicity by measuring the body weight, the weights of the main organs, and the blood indices of mice. The results showed that there was no significant variation in the body weight, the weights of the main organs, and blood indices of mice, revealing no evidence of normal tissue toxicity (**Figure 5A and Supplementary Figure S5A**). Tumor volume and weight were measured to assess tumor growth and radiosensitivity. Compared with the DMSO group, olaparib combined with ML216 significantly decreased the tumor volume of A549 xenograft mice after irradiation (*P* < 0.05) (**Figure 5B and Supplementary Figure S5B**). Compared with irradiation alone, olaparib combined with ML216 significantly reduced the tumor weight of sacrificed A549 xenograft mice after irradiation (*P* < 0.05) (**Figure 5C**). These results suggested that olaparib combined with ML216 inhibited tumor growth and enhanced radiosensitivity of A549 tumor xenografts *in vivo*. Furthermore, Ki-67 immunohistochemistry was used to indicate alterations of tumor proliferation of A549 xenografts. Olaparib combined with ML216 significantly reduced the expressions of Ki-67 protein in A549 xenografts with or without irradiation (*P* < 0.05) (**Figure 5D**). In contrast, the TUNEL assay showed that olaparib combined with ML216 induced apoptosis in the absence or presence of irradiation (*P* < 0.05) (**Figure 5E**). More importantly, the alterations of proteins involved HR repair, NHEJ repair, and cell cycle checkpoint signaling *in vivo* were consistent with those *in vitro*. Olaparib combined with ML216 significantly decreased the expressions of pBRCA1 (Ser1524), Rad50, Mre11, RPA70, and Rad51 in A549 tumor xenografts after irradiation (*P* < 0.05) (**Figure 5F and Supplementary Figure S5C**), while olaparib combined with ML216 increased the phosphorylation of DNA-PKcs (Ser2056) in A549 tumor xenografts after irradiation (*P* < 0.05) (**Figure 5F and Supplementary Figure S5C**). Additionally, olaparib combined with ML216 significantly reduced the phosphorylations of ATM (Ser1981) and ATR (Ser428) (*P* < 0.05) (**Figure 5F and Supplementary Figure S5C**). These results showed that the radiosensitization mechanisms of olaparib combined with ML216 of A549 tumors involved inhibition of tumor growth, induction of apoptosis, inhibition of HR repair, an excess of NHEJ repair, and inactivation of ATM/ATR kinases. Together, these results suggested that our results based on cellular models could be translated *in vivo*, and supported the concept that a combination of olaparib and ML216 displayed synergistic radiosensitization effects on A549 lung cancer.

## Discussion

The combination of PARP inhibitors with other therapies has focused largely on enhancing the antitumor effect of PARP inhibition by creating DNA damage or modulating DNA repair^45^. Inhibition of PARP primarily provides benefits to cancers that lack DNA repair by HR such as BRCA-mutant breast cancer. By taking advantage of pharmacologically-induced synthetic lethality, the PARP1/2 inhibitor, olaparib, has obtained approval by both the U.S. Food and Drug Administration (FDA) and the European Commission (EC) for treatment of germline BRCA-mutated advanced ovarian cancer^46^. Recently, monotherapy (olaparib) was approved by the FDA for the treatment of BRCA1/2 mutant, HR repair-deficient pancreatic cancer^47^. In contrast, cancers that are typically BRCA wild-type and proficient in HR repair make it challenging to use PARP inhibitors. Extensive studies have shown that PARP inhibition by olaparib exerted a synthetically lethal effect on HR defects, and also sensitized cancer cells with HR-proficient repair to radiation and DNA damaging agents. However, the radiosensitization effect of olaparib on A549 cells remains controversial. In 1 study, A549 cells were more resistant to both radiation and olaparib than Calu-6 cells, but olaparib still enhanced the radisosensitivity of A549 cells (SER_10_ = 1.3)^48^. In contrast, another study reported that olaparib specifically enhanced radiosensitivity in HR-proficient tumor cell lines, which did not include the A549 cell line (SER_50_ = 1.04)^49^. These results prompted us to conduct a comprehensive study on the radiation response of olaparib in NSCLC cell lines. In addition, cells from patients with Bloom’s syndrome are not hypersensitive to gamma-irradiation^25^. BLM was found to be a potential target for cancer therapy due to its synthetic lethality in a range of cancers with defects in DNA damage response^50–52^. The defect of BLM also causes PARP1 hyperactivation and increases sensitivity to PARP inhibitors^53^. We therefore sought to investigate the potential interaction and radiosensitization effects of the PARP inhibitor, olaparib, and BLM inhibitor, ML216, in NSCLC cells.

First, we tested and found that 3 NSCLC cell lines (H460, H1299, and A549) had different drug sensitivities to olaparib, with H460 cells being the most sensitive to olaparib, and A549 cells being the least sensitive to olaparib. In turn, olaparib had the best radiosensitization effect on H460 cells, followed by H1299 cells, with olaparib not being able to enhance the radiosensitivity of A549 cells. Both olaparib and ML216 enhanced the radiosensitivity of H460 (*p53*^+/+^) and H1299 (*p53*^−/−^) cells, indicating that radiosensitization occurred *via* a *p53*-independent manner. These results are consistent with previous reports that the PARP inhibitor radiosensitized tumor cell lines, independently of their *p53* status^54^. Previous reports showed that BS cells and *BLM* mutants from DT40 cells were sensitive to DNA damaging agents such as methyl methanesulfonate and ultraviolet light, which might have been due to the interaction of BLM with XRCC3^55,56^. We also detected the drug sensitivity of 3 NSCLC cell lines (H460, H1299, and A549) to ML216 and the radiosensitization effect of ML216. Our results showed that H1299 cells were the most sensitive to ML216, and ML216 had the best radiosensitization effect on H1299 cells. Whether lack of *p53* in H1299 cells made it more sensitive to BLM inhibitor remains to be determined. However, H460 cells were the least sensitive to ML216, but ML216 still enhanced the radiosensitivity of H460 cells, while ML216 had no radiosensitization effect on A549 cells. Our data provided the first demonstration that BLM inhibition by ML216 enhanced the radiosensitivity of NSCLC cell lines (H1299 and H460) *in vitro*, regardless of the *p53* status. Mechanistically, both olaparib and ML216 increased total DNA damage, delayed DSB repair, and inhibited HR repair by decreasing Rad51 loading at DNA damage sites, to enhance radiation response in H1299 and H460 cells. We further confirmed that olaparib inhibited irradiation-induced HR repair by reducing expressions of HR repair proteins, including pBRCA1 (Ser1524), Rad50, Mre11, RPA70, and Rad51.

During HR repair, BLM has both pro- and anti-recombination activities, either of which may contribute to maintenance of genomic integrity^57^. Along with the DNA2 endonuclease, BLM contributes to resection of DSBs to generate a single-stranded intermediate that is bound by RPA and Rad51. BLM also interacts with Rad51 at damaged replication forks in response to replication stress^58,59^. However, limited studies about BLM inhibitors and their biological effects have been reported. A class of isaindigotone derivatives as novel BLM inhibitors was reported to disrupt the BLM/DNA interactions and regulate HR repair by promoting accumulation of Rad51 in cancer cells^29^. In the present study our results showed that BLM inhibition by ML216 inhibited HR repair by decreasing ionizing radiation-induced Rad51 foci, suggesting that BLM may promote HR repair by regulating Rad51 recruitment at DNA damage sites in response to radiation. Additionally, ML216 also decreased the levels of HR repair proteins (Rad50, Mre11, RPA70, and Rad51) after irradiation, which further confirmed that the radiosensitization effect of ML216 on NSCLC cells was mediated by inhibiting HR repair. The increased pDNA-PKcs (Ser2056) foci formation by olaparib or ML216 in H1299 cells after irradiation suggested that both olaparib and ML216 promoted NHEJ repair.

There are few reports about the radiosensitization effect of olaparib on lung cancers, with limited reports about their mechanisms of action. In 1 study, olaparib enhanced the radiosensitivity of NSCLC cells (Calu-6 and A549 for SER_10_ = 1.5 and 1.3, respectively) by increasing persistent DSBs. Olaparib combined with fractionated radiation caused tumor regression of Calu-6 xenografts by increasing tumor vascular perfusion, which may also be beneficial for drug delivery and tumor oxygenation^48^. Also, olaparib combined with fractionated radiotherapy improves the radiobiological effects on Lewis cells and xenografts, which might be induced by promoting the formation of DSBs and upregulating the expression of Bax/Bcl-2 pro-apoptotic proteins^60^. These reports showed that radiosensitization of olaparib was associated with the induction of persistent DSBs and apoptosis in lung cancer. Besides increasing persistent DSBs, our results further suggested that the mechanism of radiosensitization caused by olaparib mainly resulted from inhibition of HR repair in olaparib-sensitive NSCLC cells (H460 and H1299). We therefore concluded that PARP promoted radiation-induced HR repair by mediating recruitment of Rad51 at DNA damage sites, phosphorylation of BRCA1 (Ser1524), and expression of Rad50, Mre11, and RPA70.

In the absence of irradiation, olaparib combined with ML216 increased total DNA damage, delayed DSB repair, and induced apoptosis in olaparib-resistant A549 cells. Consistently, olaparib combined with ML216 inhibited tumor cell proliferation and induced apoptosis *in vivo*. These results indicated that the combination of PARP and BLM inhibition by small molecule inhibitors (olaparib and ML216, respectively) exerted synthetic lethality effects, which provided a therapeutic strategy to kill olaparib-resistant tumor cells. More importantly, BLM inhibition by ML216 also increased the radiosensitivity of olaparib to A549 cancer, both *in vitro* and *in vivo*. A low dose (2 µM) of ML216 produced synergistic radiosensitization effects with olaparib in A549 cells. In addition to increasing persistent DSBs, the mechanisms of the radiosensitization effect of olaparib combined with ML216 on A549 tumors were confirmed to be associated with inhibited HR repair, an impaired cell cycle checkpoint, and induced apoptosis, which was also verified *in vivo*.

In 1 study, olaparib enhanced radiosensitivity to proton beam irradiation by disturbing the DDR, possibly by increasing the conversion of non-DSB lesions to lethal DNA damage. At 10 and 24 h after irradiation of A549 cells, olaparib induced a decrease in G1 phase and an increase in the G2/M phase cell populations, compared to irradiation^61^. In contrast, our results showed that olaparib increased G1 phase and decreased G2/M phase populations without a statistically significant change in irradiation-treated A549 cells. In addition, the ML216 phenotype increasing the G1 phase population was more obvious relative to the olaparib in irradiation-treated A549 cells. Moreover, olaparib combined with ML216 significantly increased G1 phase populations and decreased G2/M phase populations in irradiation-treated A549 cells. In particular, the reductions of G2 phase proteins further demonstrated that olaparib combined with ML216 abrogated G2 cell cycle arrest in A549 cells after irradiation. We speculated that these results were related to HR repair predominating in the S and G2 phases of the cell cycle, and with NHEJ repair dominating in the G1 phase of the cell cycle^36,40^. Also, BLM mainly functions to promote HR repair in S and G2/M phases^62,63^. Our results confirmed that interfering with 1 of these 2 DSB repair (HR and NHEJ) pathways caused a strong radiosensitizing effect^64^. Unlike highly conserved and error-free HR repair, the NHEJ pathway is error-prone and results in genomic instability^40^. Previous studies showed that PAPR inhibition enhanced error-prone NHEJ repair, which led to genomic instability and eventual cell death^53,65–67^. Consistent with these reports, we found that olaparib combined with ML216 promoted NHEJ repair in A549 tumors after irradiation *in vitro* and *in vivo*. The disruption of the balance between HR and NHEJ repair may be the most critical mechanism of the radiosensitization effect of olaparib combined with ML216 on A549 lung cancer (**Supplementary Figure S6**).

In conclusion, we have demonstrated for the first time that both olaparib and ML216 enhanced the radiosensitivities of olaparib-sensitive NSCLC cell lines (H460 and H1299) by inhibiting HR repair and promoting NHEJ repair. Targeting BLM by ML216 sensitized NSCLC to radiation and olaparib. More importantly, olaparib combined with ML216 produced a synergistic radiosensitization effect in olaparib-resistant A549 lung cancer cells, both *in vitro* and *in vivo*. Mechanistically, the combination of PARP and BLM inhibition by inhibitors (olaparib and ML216, respectively) decreased error-free HR repair, promoted error-prone NHEJ repair, inactivated cell cycle checkpoints, accumulated DSBs, and eventually caused apoptotic cell death. These observations provided a novel strategy for future studies directed toward overcoming radio- and olaparib-resistance in NSCLC and developing therapeutic treatment by targeting PARP and BLM as biomarkers.
