# Supplementary material for: BLM helicase inhibition synergizes with PARP inhibition to improve the radiosensitivity of olaparib resistant non-small cell lung cancer cells by inhibiting homologous recombination repair
Source: Cancer Biol Med. 2021 Dec 1;19(8):1150–71. doi: 10.20892/j.issn.2095-3941.2021.0178 (PMC9425185; doi:10.20892/j.issn.2095-3941.2021.0178)
Supplement: Supplementary file 1 [file cbm-19-1150-s001.pdf]

Supplementary materials

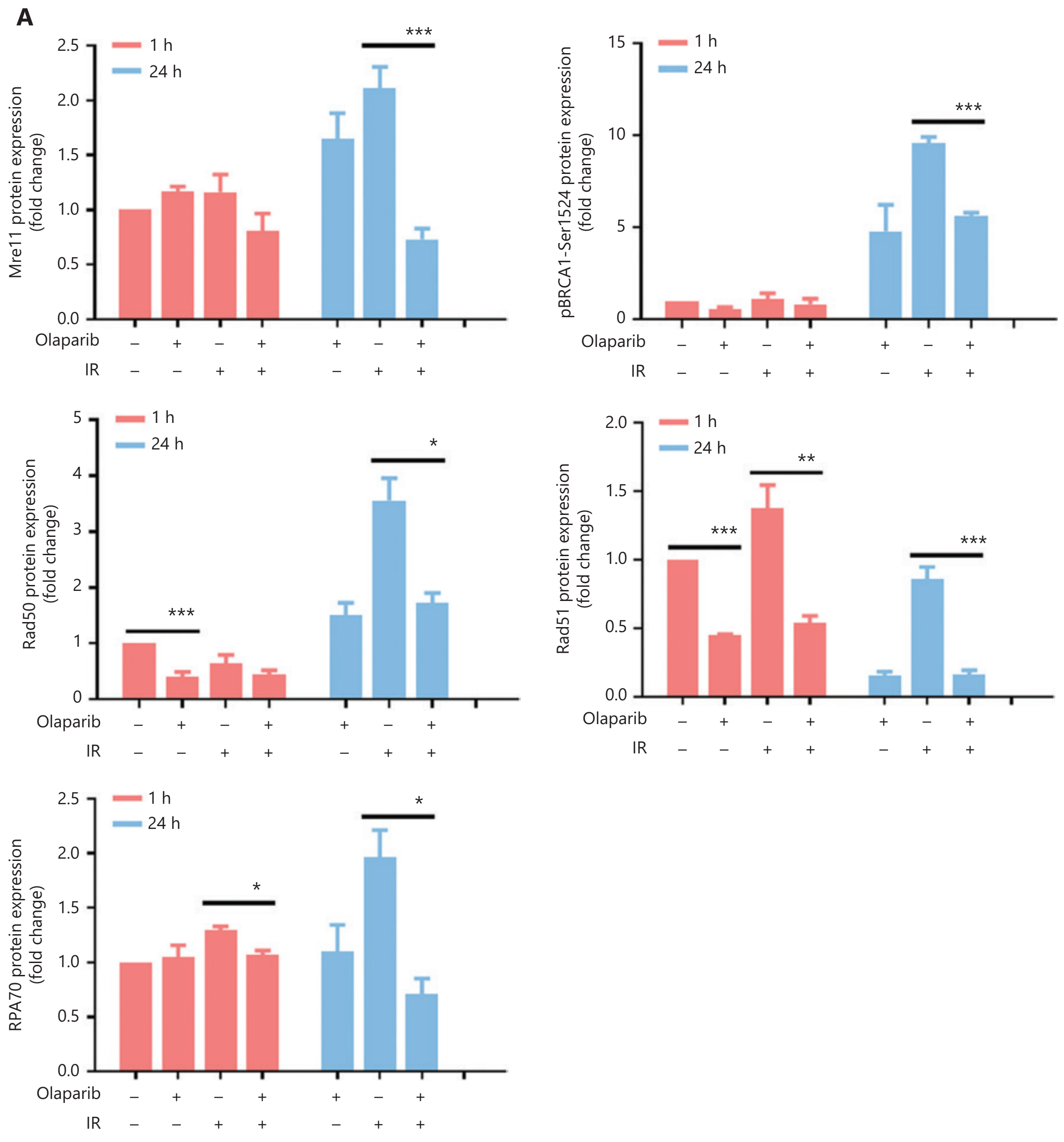

Figure S1 Continued

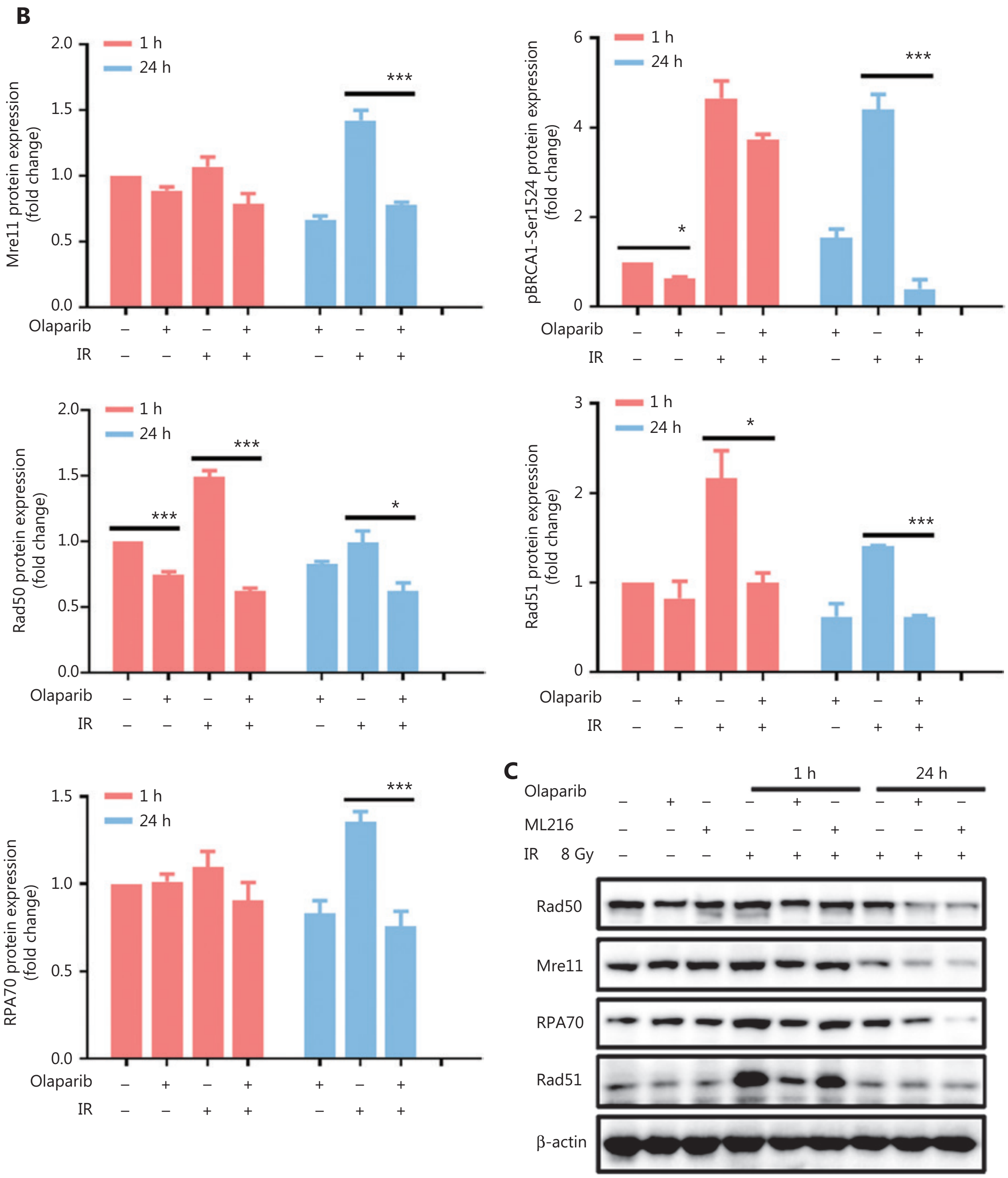

Figure S1 Both olaparib and ML216 reduced the protein levels of homologous recombination repair in H460 and H1299 cells after irradiation. A statistical chart of Western blot experiments of H460 cells (A) and H1299 cells (B) in **Figure 3**. (C) H1299 cells treated with dimethyl sulfoxide, 5 μM olaparib, or 10 μM ML216 were harvested at 1 h and 24 h, after 8 Gy of irradiation. The cells were lysed in protein extraction buffer and then denatured at 100 °C for 10 min. The protein samples were resolved by SDS-PAGE and analyzed by ImageJ Lab software. Shown are the means ± SEM from 3 experiments (*P < 0.05; **P < 0.01; ***P < 0.005).

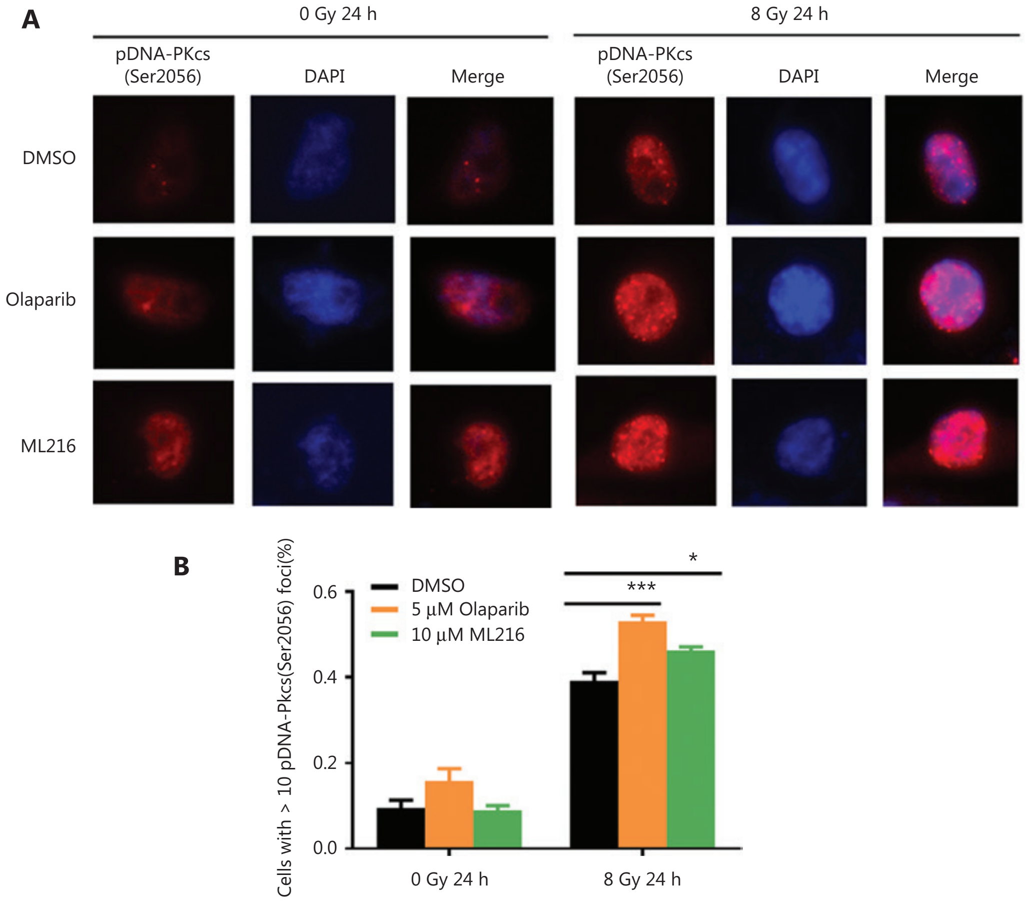

Figure S2 Both olaparib and ML216 increased the pDNA-PKcs (Ser2056) foci formation in H1299 cells. H1299 cells treated with dimethyl sulfoxide, 5 µM olaparib, or 10 µM ML216 were fixed in paraformaldehyde 24 h after 8 Gy of irradiation. The cells with more than 10 foci per cell were regarded as positive cells, and at least 300 cells were counted. The ratio of positive cells and representative pictures are shown. Shown are the means ± SEM from 3 experiments (*$P < 0.05$; ***$P < 0.005$).

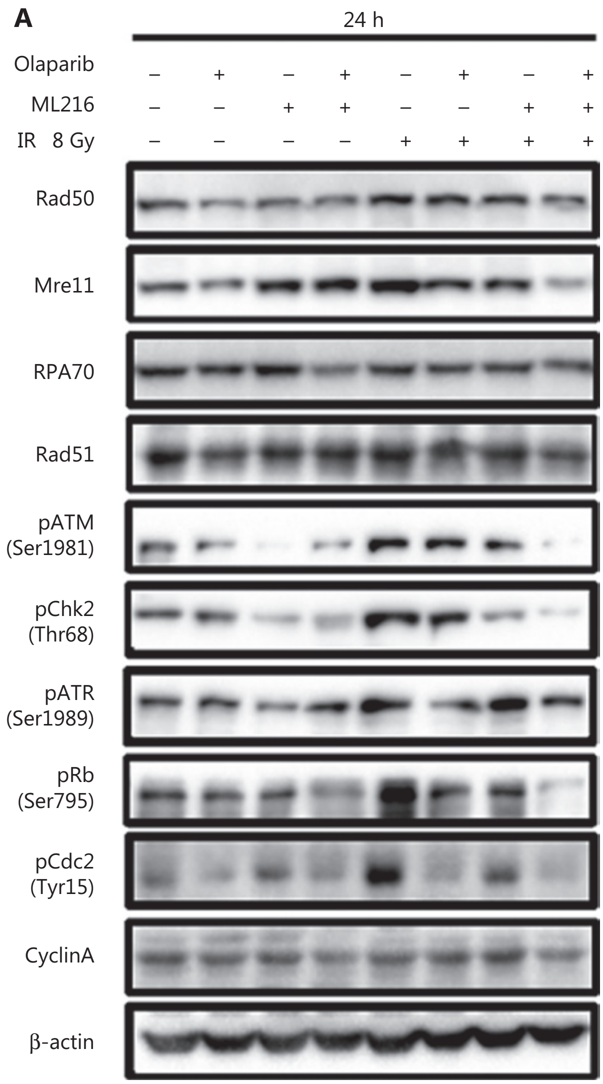

Figure S3 Continued

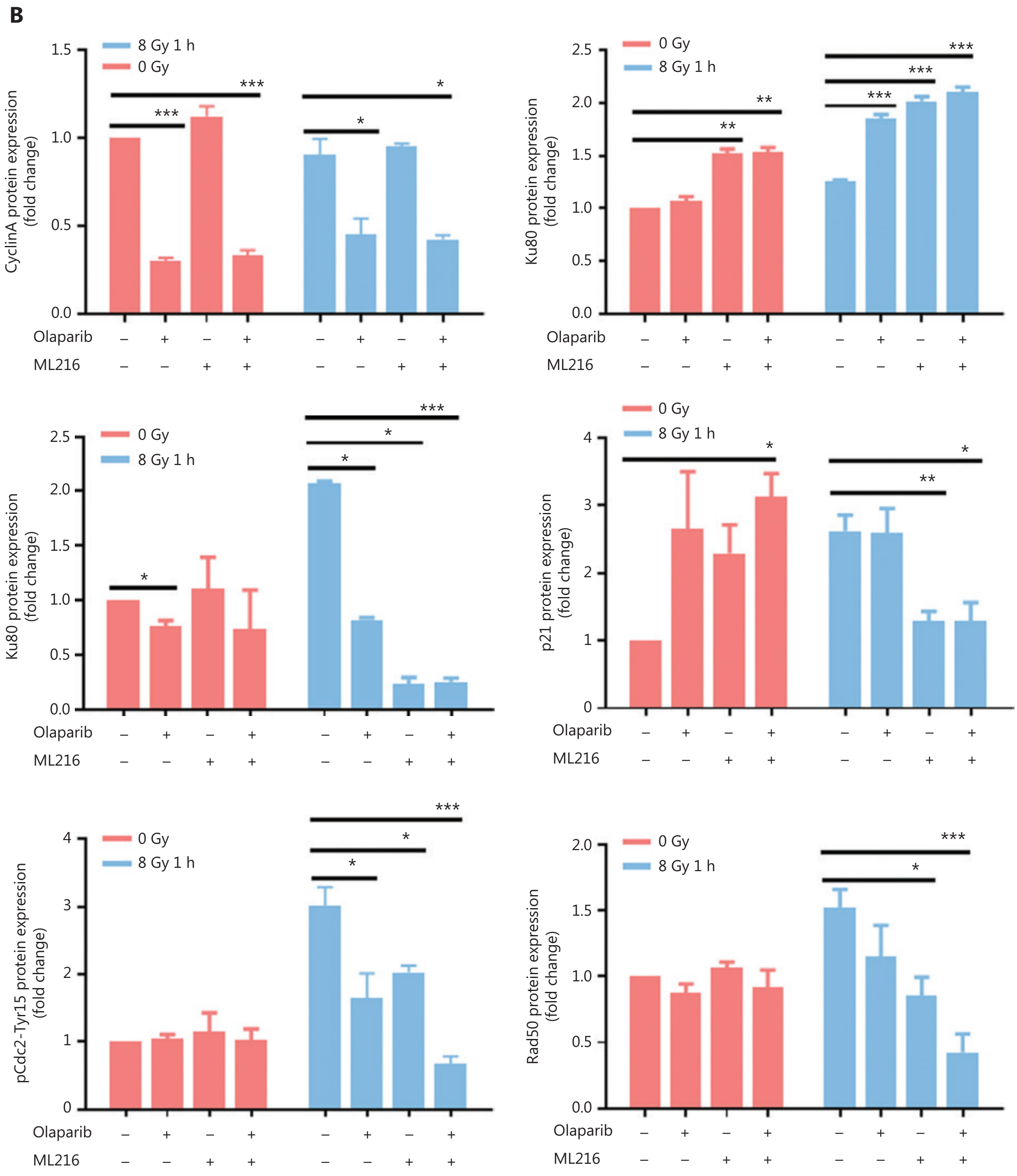

Figure S3 Continued

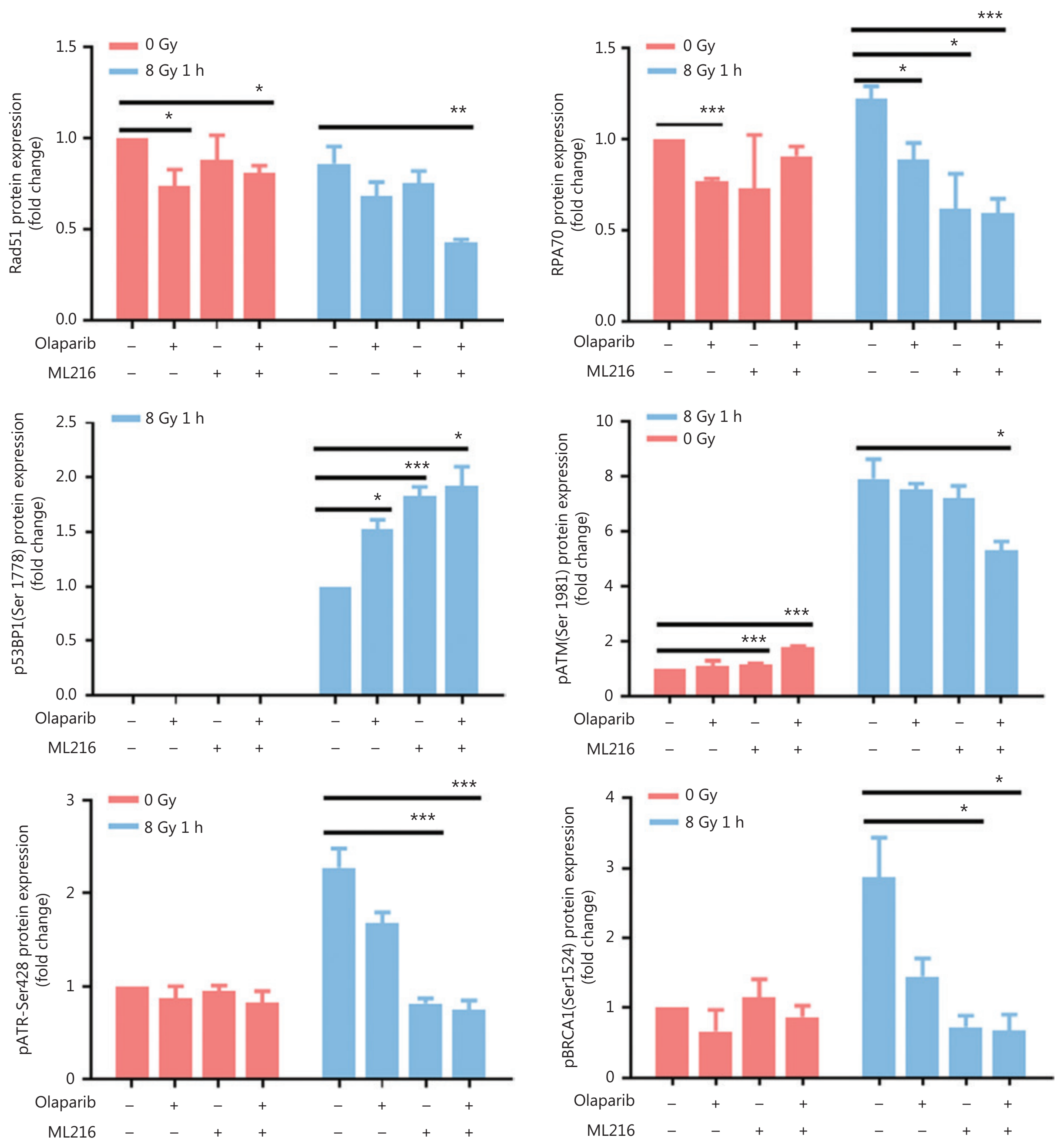

Figure S3 Continued

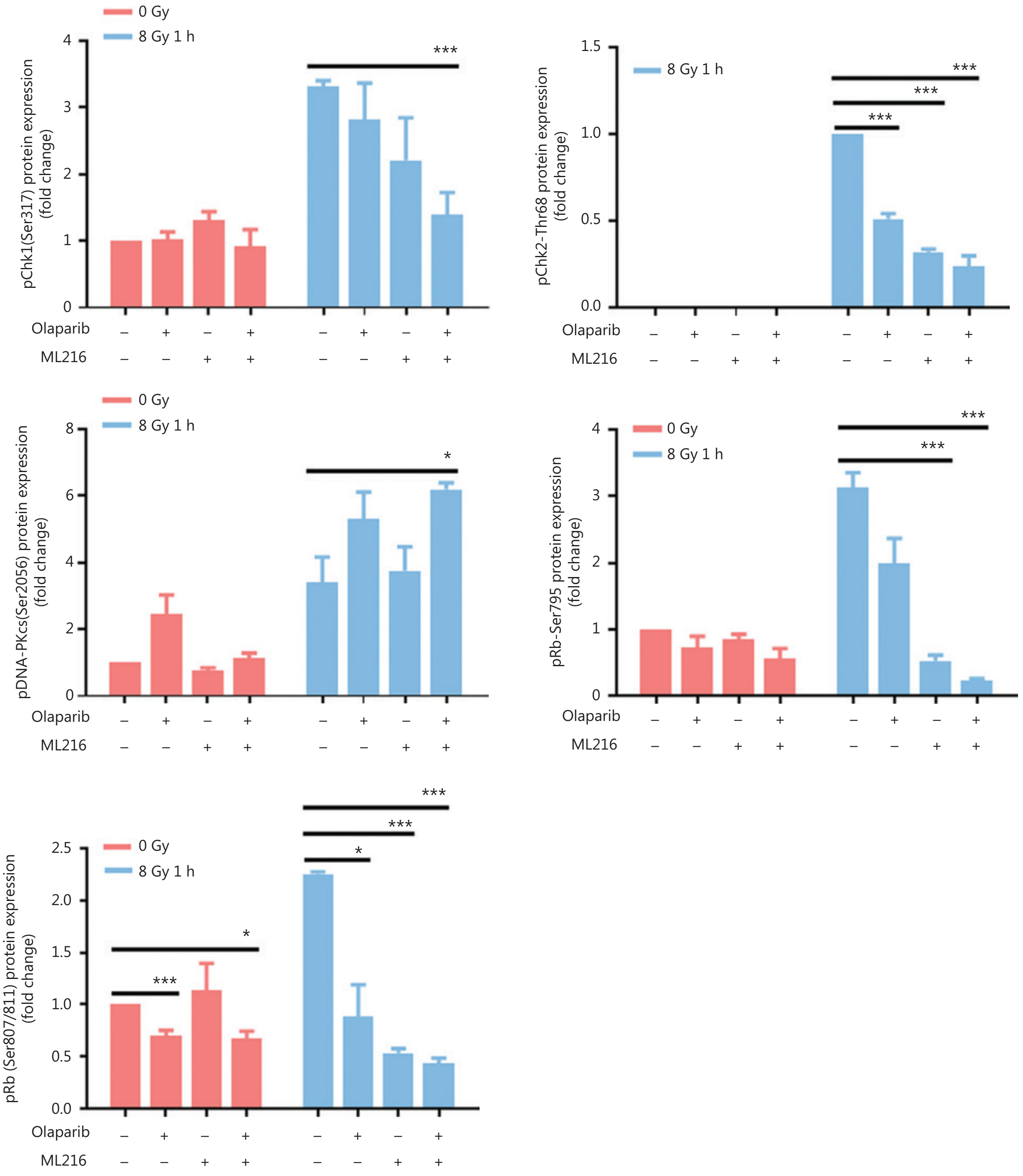

Figure S3 Olaparib combined with ML216 decreased the protein levels of homologous recombination repair, DNA damage response recognition, and G2 cell cycle arrest in A549 cells after irradiation. (A) A549 cells treated with dimethyl sulfoxide, 5 μM olaparib, or 10 μM ML216 were harvested at 24 h after 8 Gy of irradiation. The cells were lysed in protein extraction buffer and then denatured at 100 °C for 10 min. The protein samples were resolved by SDS-PAGE and analyzed by ImageJ Lab software. Representative pictures of Western blot are shown. (B) The statistical chart of the Western blot experiment of A549 cells in **Figures 3 and 4**. Shown are the means ± SEM from 3 experiments (*$P < 0.05$; **$P < 0.01$; ***$P < 0.005$).

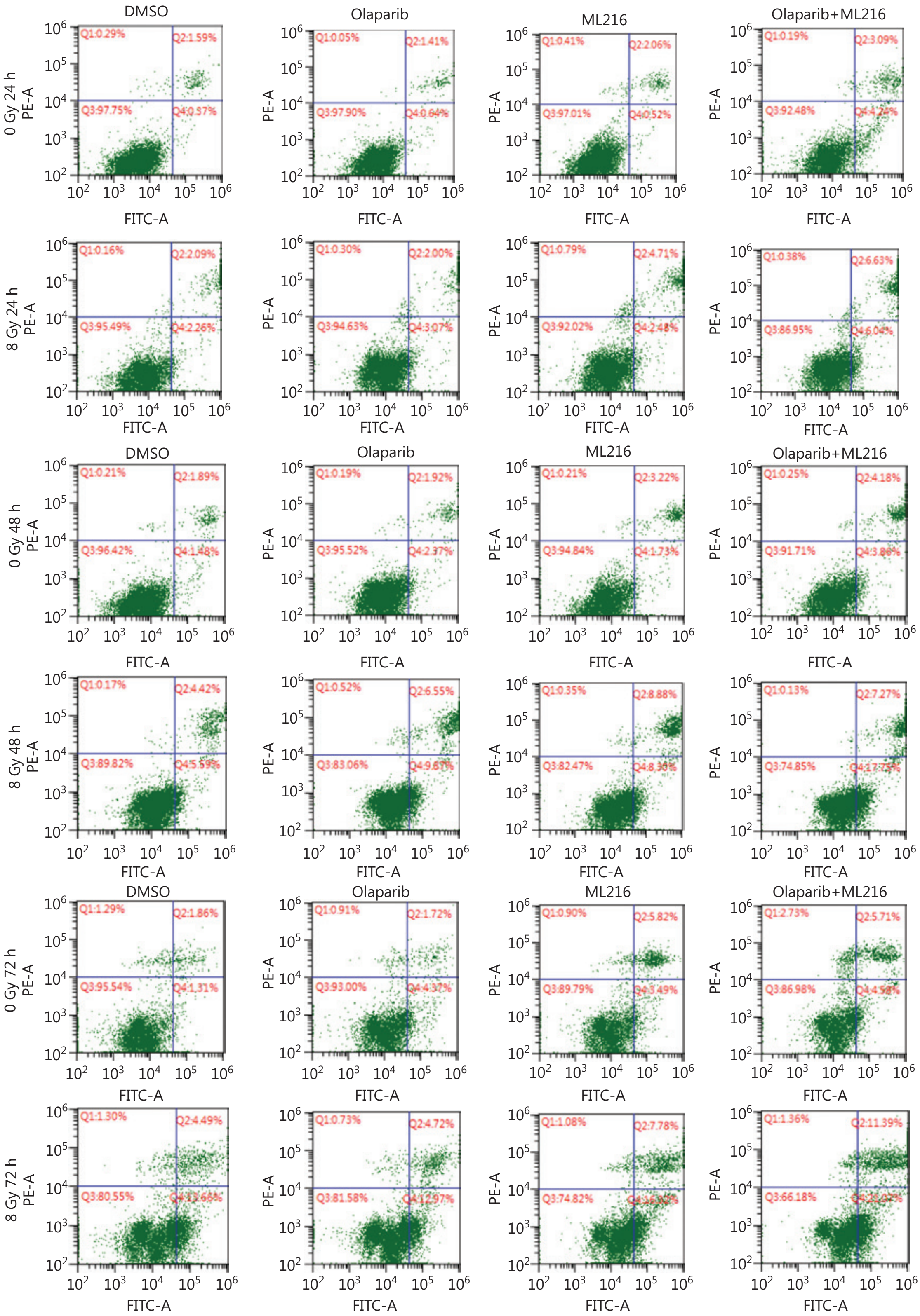

Figure S4 Representative pictures of apoptosis in A549 cells by flow cytometry. The 4 quadrants of the dual parameter fluorescent dot plots represent different states of the cells. The viable cell population was in the third quadrant (PI$^-$/FITC$^-$). The early apoptotic cells were in the fourth quadrant (PI$^-$/FITC$^+$). The late apoptotic and necrotic cells were in the second-quadrant (PI$^+$/FITC$^+$).

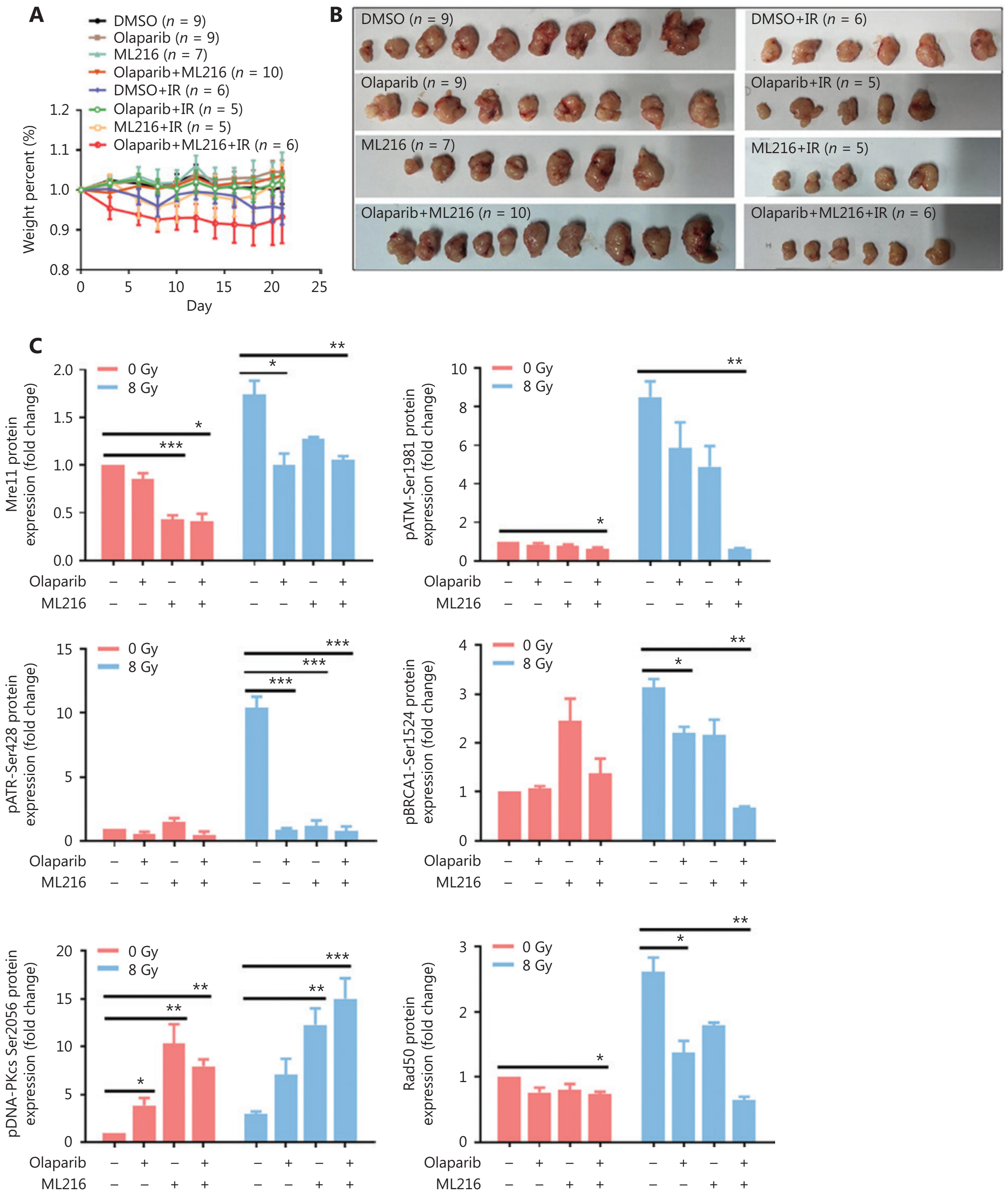

Figure S5 Continued

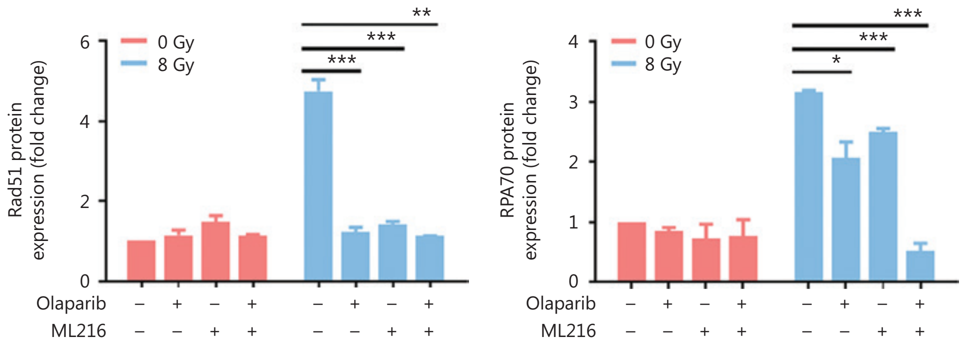

Figure S5 The radiosensitization effect of olaparib combined with ML216 of A549 xenografts. (A) The body weights of mice were measured for 21 days. (B) Pictures of A549 xenograft tumors. (C) The statistical chart of Western blot experiments of A549 xenograft tumors in **Figure 5**. Shown are the means ± SEM from 3 experiments (*P < 0.05; **P < 0.01; ***P < 0.005).

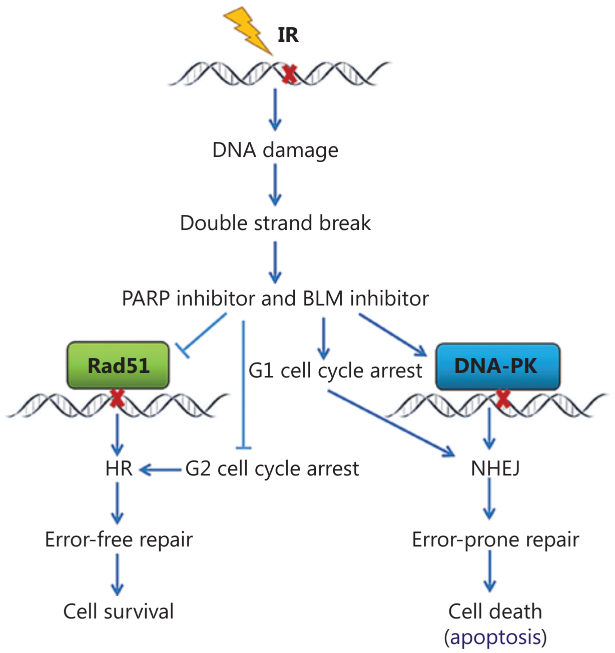

Figure S6 Radiosensitization mechanism of olaparib and ML216 in non-small cell lung cancer (NSCLC) cells. The imbalance of double strand break (DSB) repair pathways, manifested as inhibiting error-free homologous recombination repair and promoting error-prone non-homologous end joining repair, leading to DSB accumulation, finally induced apoptosis and enhanced the radiosensitivity of NSCLC cells.

Table S1 Inhibitor concentrations to produce 50% inhibition (IC_{50})

Cell lines	Inhibitor	Days	IC_{50} (μM)
H460	Olaparib	3	3.461
		5	1.908
	ML216	3	2.782
		5	2.139
H1299	Olaparib	3	2.240
		5	2.902
	ML216	3	2.615
		5	3.827
A549	Olaparib	3	11.92
		5	5.948
	ML216	3	9.036
		5	2.100
